# STAT3 roles in viral infection: antiviral or proviral?

**DOI:** 10.2217/fvl-2018-0033

**Published:** 2018-07-02

**Authors:** Zhangmei Chang, Yan Wang, Xin Zhou, Jian-Er Long

**Affiliations:** 1 Key Laboratory of Medical Molecular Virology of Ministries of Education & Health, Shanghai Medical College of Fudan University, Shanghai 200032, PR China; 2 Department of Medical Microbiology & Parasitology, Laboratory of Medical Microbiology, Shanghai Medical College of Fudan University, 138 Yixueyuan R., Shanghai 200032, PR China; 3 Kunshan Center For Disease Control & Prevention, 458 Tongfengxi Road, Kunshan, Jiangsu, 215301, PR China

**Keywords:** STAT3, viral infection, JAK/STAT

## Abstract

Signal transducer and activator of transcription 3 (STAT3) is a transcription factor which can be activated by cytokines, growth factor receptors, and nonreceptor-like tyrosine kinase. An activated STAT3 translocates into the nucleus and combines with DNA to regulate the expression of target genes involved in cell proliferation, differentiation, apoptosis and metastasis. Recent studies have shown that STAT3 plays important roles in viral infection and pathogenesis. STAT3 exhibits a proviral function in several viral infections, including those of HBV, HCV, HSV-1, varicella zoster virus, human CMV and measles virus. However, in some circumstances, STAT3 has an antiviral function in other viral infections, such as enterovirus 71, severe acute respiratory syndrome coronavirus and human metapneumovirus. This review summarizes the roles of STAT3 in viral infection and pathogenesis, and briefly discusses the molecular mechanisms involved in these processes.

Signal transducer and activator of transcription factors (STATs) are a family of transcription factors that regulate cell proliferation, differentiation, apoptosis, inflammation and oncogenesis [1–3]. Seven STAT proteins (STAT1–4, STAT5a, STAT5b and STAT6) have been discovered in mammals [2]. When activated by cytokines and growth factors, STATs become phosphorylated on specific tyrosine residues by Janus kinases (JAKs) and form homodimers or heterodimers and then translocate to the nucleus where they regulate the transcription of target genes [3]. Each STAT protein contains six conservative domains; however, upon activated by the specific stimulators, the STATs are phosphorylated in a STAT-specific manner at distinct residues in the C-terminal domain by different JAK members. Therefore, STATs play different roles in the multiple biological processes [4,5]. Specifically, STAT3 plays a central role in the development, immunity and carcinogenesis, since it critically regulates the transcription of multiple key genes involved in cell proliferation, differentiation, apoptosis, immune responses, angiogenesis and metastasis [5].

STAT3 is first identified as a DNA-binding factor that selectively attaches to the IL-6-responsive elements in the promoter region of the IL-6-stimulated hepatocytes under the acute phase [6]. As a central axis signal transduction molecule, STAT3 plays an important regulatory role in the process of cellular immune responses, antagonizes the release of cytokines, such as IL-12 and IFN-γ, from Th1 cell, and regulates the development and differentiation of T helper cells, such as T_reg_, Th_1_ and Th_17_ [7–9]. Furthermore, it can regulate cell apoptosis through the *Fas, BCL-XL*, and *BCL-2* genes [10]. Recently, the role of STAT3 in viral infection has been attracting research interest. Several studies showed that STAT3 affects many kinds of viral infections and pathogeneses, including those of HBV, HCV, varicella zoster virus (VZV), and severe acute respiratory syndrome coronavirus (SARS-CoV) [11–14]. The present review summarizes the roles of STAT3 in these viral infections and pathogeneses, and simply discusses the molecular mechanisms by which STAT3 functions, specifically in the virus life cycles and related disease development.

## Structure of STAT3 protein

**Figure 1.**
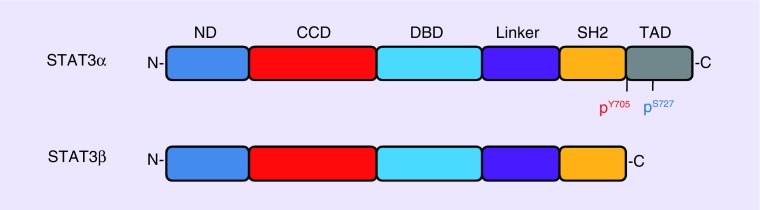
**Schematic structure of STAT3.** STAT3 has two isoforms, STAT3α and STAT3β. STAT3α protein is composed of ND, CCD, DBD, linker domain, SH2 domain and a C-terminal TAD. However, the transactivation domain is absent in the alternative splicing variant, STAT3β. CCD: Coiled-coil domain; DBD: DNA binding domain; ND: N-terminal domain; TAD: Transcriptional activation domain.

The human gene that encodes STAT3 is located on chromosome 17q21. The STAT3 protein is 84–113 kDa and contains 750–795 amino acids [15]. Like other STATs, The STAT3 protein comprises six functional regions, namely, N-terminal domain, coiled-coil domain, DNA binding domain, linker domain, SH2 domain and a C-terminal transcriptional activation domain (TAD) (Figure 1). N-terminal domain is involved in STAT3 dimerization, and coiled-coil domain is associated with important regulatory modifiers. DNA binding domain forms complexes between STAT3 and DNA, and the SH2 domain recruits two activated STATs to form dimers. TAD, which communicates with transcriptional complexes, contains a tyrosine residue at position 705 and a serine residue at position 727, both of which can be phosphorylated after activation [16]. STAT3 possesses two isoforms, namely, STAT3α and STAT3β (Figure 1). STAT3α is the main isoform expressed in cells. In contrast to STAT3α, STAT3β is truncated in TAD by alternative splicing. STAT3β can inhibit the transactivation effects of STAT3α by competitively binding to the promoter of STAT3α target genes [17].

## Activation of STAT3 signaling

**Figure 2.**
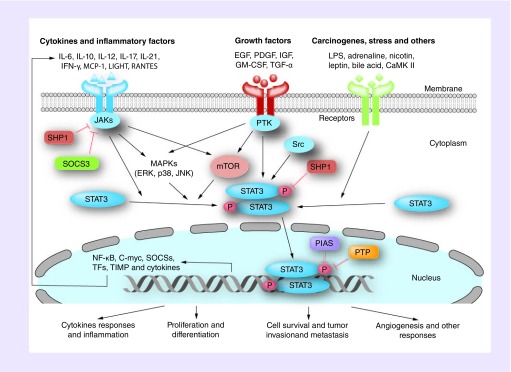
**STAT3 activation and signaling regulation.** In response to some cytokines, growth factors and other triggers, STAT3 is phosphorylated by receptor-associated kinases and then forms homo- or heterodimers. STAT3 dimers then translocate to the nucleus where they act as a transcription activator and mediate the expression of a variety of genes. These genes play important roles in inflammation, oncogenesis, cell survival and angiogenesis. It should be noticeable that some key components for STAT3 signaling are regulated by several phosphatases and suppressors. Please see the detail in the text. Arrows indicated the increase, whereas T-line means the blockage. PIAS: Protein inhibitor of activated STAT; SOCS: Suppressor of cytokine signaling.

The STAT3 protein exists in an inactive form in the cytoplasm. STAT3 is activated by several cytokines including the IL-6 family, cardiotrophin-1, ciliary neurotrophic factor (CNTF), IL-5, IL-9, IL-10, IL-11, IL-12, IL-21, IL-22, IL-27, LIGHT, MCP-1, TNF-α and IFN-γ [10]. These cytokines bind to their specific receptors to form oligomerized receptor complexes, alter the cytoplasmic domain and activate the corresponding JAK. The JAKs, in turn, phosphorylate the specific tyrosines in the intracellular domains of the receptors, while recruiting and phosphorylating STAT3. The activated STAT3 separates from the receptor to form homodimers or heterodimers with other STAT proteins through SH2 domain interaction. Dimerized STAT3 is then translocated from the cytoplasm to the nucleus to bind to the target gene promoter region and induces gene expression (Figure 2) [18].

STAT3 can also be activated by various growth factor receptors, including EGF receptors (EGFRs), HGF receptors, FGF receptors, PDGF receptors, and IGF receptors (IGFRs) [19] and by hormones and oncogenes through receptor tyrosine kinases (Figure 2) [20].

STAT3 signaling is activated by phosphorylation in the C-terminal domain. Tyr705 phosphorylation is mainly induced and required for dimerization to activate STAT3 signaling. The phosphorylation of STAT3 at serine 727 is mediated by serine kinases as observed in the mTOR and MAPKs including ERK, p38 and JNK, and leads to the upregulation of the transcriptional activity [21]. Unphosphorylated STAT3 (U-STAT3) can also communicate with NF-κB to form a complex, which then translocates into the nucleus and activates the expression of NF-κB-regulating genes [22].

Besides being phosphorylated at tyrosine 705 and serine 727, STAT3 is also acetylated at lysine residue 685 by histone acetyltransferase p300 [23]. The process is inhibited by histone deacetylases (HDACs). Acetylated STAT3 (Ac-STAT3) participates in forming stable dimmers [23].

## Regulation of STAT3 signaling

STAT3 activation of is generally rapid and transient and plays a key role in the physiological function of normal cells. STAT3 activation is regulated by mechanisms involving suppressor of cytokine signaling (SOCS), protein inhibitor of activated STAT (PIAS), protein tyrosine phosphatases and genes associated with the retinoid-IFN-induced mortality (GRIM-19) [13,14,18]. The SOCS3 can inhibit STAT3 phosphorylation by binding to the SH2 domain of JAKs and consequently blocks the subsequent phosphorylation of STAT3 [13], and PIAS-3 inhibits the DNA-binding of STAT3 by binding to the STAT3 dimer (Figure 2) [14].

## Function of STAT3

STAT3 is widely expressed in various tissues and involved in cell proliferation, differentiation, apoptosis and oncogenesis (Figure 2). STAT3-knockout mice are embryonic lethal [24], and STAT3 maintains the self-renewal of embryonic stem cells [25]. In the immune system, STAT3 affects T- and B-cell maturation, differentiation and function. The differentiation of CD4^+^ T cells (including Th17 and Th2 cells) is dependent on STAT3 [26–29]. In brain cells, STAT3 can be activated by CNTF receptor and leptin. CNTF is incapable of inducing the phosphorylation of protein kinase B (Akt and PKB) in STAT3-deficient neurons, indicating that STAT3 has a direct effect on Akt activation [30]. Furthermore, STAT3 activation is critical to the differentiation of keratinocytes [31]. Mice lacking STAT3 activity in keratinocytes showed significantly disrupted regrowth and wound repair [32]. Additionally, STAT3 is specifically activated during mammary gland cell degeneration, and the absence of STAT3 in mammary cells causes a delayed involution of the mammary glands [33]. Given the diversity of STAT3 functions, many viral infections are associated with the STAT3 signaling.

## Role of STAT3 in viral infection

**Figure 3.**
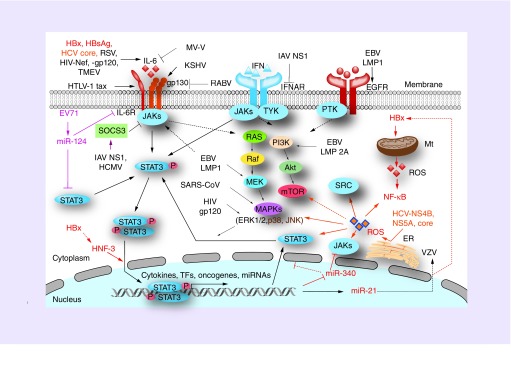
**STAT3 signaling pathway modulated by the virus infections.** The viruses mainly modulate STAT3 signaling through the IL-6/IL-6R/gp130/JAKs cascade. STAT3 signaling, sometimes triggered by other cytokines, growth factor and tyrosine kinase, are also modified by several viruses. Furthermore, the downstream components of the IL-6/STAT3 cascade can interact with the Ras/Raf/MEK/MAPKs and PI3K/Akt/mTOR pathways. The details about how the viruses regulate STAT3 signaling can be seen in the main text. Main pathways modified by the viruses are indicated with the bold arrow; the broken line indicates that the effects are mainly mediated by in an indirect way. The HBV- and HCV-induced effects on STAT3 signaling are specifically indicated in the red and orange colors, respectively. ER: Endoplasmic reticulum; Mt: Mitochondria; RABV: Rabies virus; ROS: Reactive oxygen species; RSV: Reproductive and respiratory syndrome virus; SOCS: Suppressor of cytokine signaling; TF: Transcription factor; TMEV: Theiler's murine encephalomyelitis virus.

STAT3 can be activated by IL-6 and IL-10 family cytokines and growth factors [10]. Thus, STAT3 plays a key role in the pathogenesis of many cancers [34], lymphocyte differentiation and innate immune response [35,36]. However, the role of STAT3 in viral infection is complicated, and various viruses cause differences in the host cell STAT3 activation (Table 1 & Figure 3). STAT3 participates not only in host antiviral immune response, but also in the inflammatory responses induced by viruses. Thus, the role of STAT3 in virus replication is uncertain and seems to function as a proviral or antiviral factor in a virus-specific manner (Table 1).

**Table 1.** **Dual role of STAT3 in viral infection.**

**Roles in viral infection**	**Research models**	**Molecular mechanism**	**Ref.**
**Proviral activity or promote the virus-induced pathogenesis**			

HBV	HepG2 cells	HBx activates STAT3 signaling through oxidative stress	[37]

	Human hepatoma cell lines (Hep3B and PLC/PRF/5) and nontumourigenic hepatocyte cells (MIHA and L02)	HBx upregulates miR-21 mediated by IL-6/STAT3 pathway	[38]

	HBV transgenic mice	HBsAg impairs T-cell activation by polarizing monocytes toward mMDSCs in an ERK/IL-6/STAT3 signaling-dependent manner	[39]

	Clinical HCC specimens and cell lines (HepG2, HepG2.2.15, HuH7)	HBV downregulates miR-340-5p to induce STAT3 and promote the migration of liver cancer cells	[40]

	HepG 2.2.15	STAT-3 and HNF-3 binding cooperatively stimulates the HBV enhancer I function in response to IL-6 and EGF	[14]

	HBV transgenic mice and HepG 2.2.15	RT and IL-6 induced HBV reactivation through STAT3 signaling	[41]

	The human HCC cell lines Bel-7402 and SMMC-7721	HBx-ΔC1 enhances liver cancer stem cells properties through Stat3/Nanog cascade	[42]

	Patients with HBVGN and HK-2 cells	HBx upregulates the Bax/Bcl-2 ratio by activating JAK2/STAT3 to cause renal tubular epithelial cell apoptosis	[43]

HCV	Huh 7 cells	NS5A induces oxidative stress and activates STAT3 and NF-κB	[44]

	Huh-7 and FCA4 cells and liver biopsy specimens	HCV activates STAT3 through oxidative stress and induces the activation of tyrosine and MAP kinases	[45]

	Purified monocytes from healthy donors	Extracellular HCV core protein activates STAT3 through PI3K/Akt and IL-6 pathway, which impairs T-cell response	[46]

	Mice expressing HCV core and	Causes ROS and lowers mitochondrial transmembrane potential, leads to DNA damage and activation of STAT3	[47]

	TRNS3/4A cell		

	Huh-7, Huh-7.5, NNeoC5B and NNeo3-5B cells	Activation of STAT3, and which positively regulates MT dynamics to increase HCV replication	[11]

	Huh7	STAT3 upregulates lnc-IGF2-AS and lnc-7SK through PI4P to promote HCV replication	[48]

	Huh7	HCV upregulates STAT3 by sponging miR-122 and inhibits the expression of IFN-α and IFN-β	[49]

	Purified monocytes from healthy donors	HCV core induces MDSC-like monocytes via TLR/PI3K/AKT/STAT3 signaling to inhibit CD4^+ ^T-cell activation	[50]

	HepG2 and NIH 3T3 cells	Activation of STAT3 results in rapid proliferation and upregulation of Bcl-XL and cylin-D1	[51]

	HepG2 and Huh7	NS5A activates STAT3 signaling through Jak1, and then upregulates the STAT3 downstream molecules Bcl-xL and p21	[52]

	HepG2	HCV core induced STAT3 phosphorylation and upregulates NANOG	[53]

	Huh-7, Huh7.5.1, and PHHs	NS4B activates STAT3, which stimulates cancer-related target genes expression	[54]

VZV	HELF and human skin xenograft in SCID mice	Activation of STAT3, by which to increase the expression of survivin	[13]

	MeWo and HELF cells	Increases in miR-21 and activation of the STAT3 signaling	[55]

HCMV	MRC-5, ARPE19 and U373 cells	HCMV utilizes unphosphorylated STAT3 to promote HCMV DNA replication, IE1 as a regulator of STAT3 intracellular localization and IL-6 signaling	[56]

	HepG2 and PHH cells	Activation of IL-6/JAK/STAT3, which enhances the rumor sphere formation	[57]

EBV	NPC cell line (CNE1)	LMP1 stimulated STAT3 via JAK3 and ERK	[58]

	Cervical carcinoma cell line C33A cells	LMP1-CTAR1 activates STAT3, which increase Bcl-3 to regulate EGFR	[59]

	Cervical carcinoma cell line C33A cells, Rat-1 cells and 293T cells	LMP1 activates EGFR, STAT3 and ERK via PKCδ	[60]

	HeLa and HEK 293T	EBNA2 augments STAT3-DNA binding ability	[61]

	B-cell lines	LMP2A activates STAT3 via PI3K/BTK pathway	[62]

	NPC cell lines, HONE-1 and CNE-2	Activation of STAT3 directly contributes to the intrinsic invasiveness of NPC cells	[63]

	Human NPC cell line CNE1	LMP1 activates STAT3 contributes to the invasion of NPC	[64]

	CNE1	LMP1-activated EGFR and STAT3 promote cyclin D1	[65]

	Patient samples, NPC cell lines (HNE-1, 5-8F, HONE-1, C666-1)	LMP-1 suppressed miR-204 expression by activating STAT3	[66]

	P3J-HR1K BL cells (HH514-16)	STAT3 presents high level in the refractory state	[67]

	Patients and EBV-infected HH514-16 cell line	STAT3 upregulates KRAB-ZFPs, and limits lytic activation to favor the persistence of tumor cells	[68]

	EBV lymphoblastoid cell lines (EBV-LCLs)	STAT3 regulates the lytic susceptibility via cellular PCBP2	[69]

KSHV	TIME cells, hDMVEC cells, BCBL-1 and BJAB cells	Activation of STAT3 by gp130 and JAK2	[70]

RRSV	A549, HEP-2 cells	Activation of STAT3 via IL-6	[71]

HIV	Immature DCs	Nef activates STAT3	[72]

	Primary human monocyte/macrophages	Nef activates STAT3 through the release of soluble factors	[73]

	Immature MDDCs	Gp120 activates IL/STAT3 pathway	[74]

HTLV-1	T-cell lines	Tax activates sIL-6R/STAT3, which enhanced proliferation of HTLV-I-infected T cells	[75]

	HTLV-1-infected patients sample and T-cell lines	IL-10 mediates lymphoproliferation by STAT3 and IRF4 pathways	[76]

DV	DCs, HepG2	Activation of JAK2/STAT3 induces chemokine production	[77,78]

TMEV	Female C57BL/6 (B6) mice, IL-6 KO mice Human IL-6 Tg mice	Increased IL-6 promotes IL-17, they activate STAT3 and NF-κ B to promote viral persistence	[79]

**Antiviral activity or inhibits the virus-induced pathogenesis**			

HSV	Murine model	Phosphorylated STAT3 activates genes including Bcl-2, Bcl-XL, IAP2, Pim-1, neurodegenerative GAP-43 and neuronal development GFPAF, to maintain the latent state of HSV	[80]

	C57BL/6 mice	IL-6-induced STAT3 cascade prevents the loss of neuronal precursors induced by HSV-1	[81]

	STAT3-knockout mice and Vero cells	STAT3-knockout cells were more susceptible to HSV-1	[82]

IAV	HeLa	H5N1 NS1 reduce IFN-induced STAT3	[83]

	Huh7 and A549	STAT3 induce ISGs to inhibit IAV replication	[84]

	Human type I-like alveolar epithelial cells	H5N1 and H1N1 inhibit the activation of STAT3	[85]

Mumps virus	Human 2FTGH, 293T, NIH 3T3, 3T3/v-Src, U3A and U6A cell lines	V protein causes STAT3 degradation and prevents responses to IL-6 and v-Src	[86]

Measles virus	Human 2fTGH, 293T and 293 Tet-On cell lines, and murine NIH 3T3 cells	V causes a defect in IFN-induced STAT nuclear accumulation, including STAT3, to prevent responses to IL-6 and v-Src	[87]

SARS-CoV	Vero E6 cells	Activation of p38 MAPK pathway induces STAT3 dephosphorylation to promote replication	[88]

hMPV	A549 and monkey kidney cells	Attenuates the IL-6-mediated JAK/STAT3 signaling cascade	[89]

PRRSV	MARC-145, HEK-293, HeLa	Nsp5 induces STAT3 degradation	[90]

CVB3	Mice with the cardiomyocyte-restricted STAT3 deletion	STAT3 in cardiomyocytes protects the CVB3-induced myocarditis by increasing the expression of collagen I and decreasing matrix degradation	[91]

EV71	RD and SH-SY5Y	EV71 increases miR-124 to inhibit IL-6R and STAT3	[92]

RABV	COS-7	RABV P protein inhibits STAT3 nuclear accumulation and gp130-dependen signaling	[93]

BL: Burkitt lymphoma; CVB3: Coxsackie virus B 3; DC: Dendritic cell; DV: Dengue virus; EGFR: EGF receptor; EV71: Enterovirus 71; HBsAg: Hepatitis B surface antigen; HBVGN: HBV-associated glomerulonephritis; HCC: Hepatocellular carcinoma; HCMV: Human cytomegalovirus; HELF: Human embryonic lung fibroblast; hMPV: Human metapneumovirus; HTLV-1: Human T-cell leukemia virus type-1; IAV: Influenza A virus; KO: Knock-out; KSHV: Kaposi's sarcoma herpesvirus; LCL: Lymphoblastoid cell lines; MDDC: Monocyte-derived DC; mMDSC: Monocyte toward myeloid-derived suppressor cell; MT: Microtubule; NPC: Nasopharyngeal carcinoma; PHH: Primary human hepatocyte; PRRSV: Porcine reproductive and respiratory syndrome virus; RABV: Rabies virus; RD: Rhabdomyosarcoma cell; ROS: Reactive oxygen species; RRSV: Reproductive and respiratory syndrome virus; RSV: Reproductive and respiratory syndrome virus; RT: Radiotherapy; SARS-CoV: Severe acute respiratory syndrome coronavirus; SCID: Severe combined immune deficiency; TMEV: Theiler's murine encephalomyelitis virus; VZV: Varicella zoster virus.

### STAT3 in hepatitis B & C virus infection

STAT3 can be activated by HBV, HCV and several oncogenes [37,44,46]. HBV or HCV infection is the major risk factor of hepatocellular carcinoma (HCC) [94]. The constitutive activation of STAT3 has been detected in HCC tissue [36].

#### Role of STAT3 in HBV infection

HBV infection is an important cause of hepatitis, cirrhosis and liver cancer. HBV-induced primary liver cancer, advanced liver cancer recurrence and metastasis are associated with persistent inflammation accompanying the expression of STAT3. HBV infection activates STAT3 through the HBV X protein (HBx) and hepatitis B surface antigen (HBsAg), and the activated STAT3 interacts with different target genes, causing numerous effects on host antiviral immunity, viral replication and HCC [37,39].

HBx is a multifunctional protein that regulates gene transcription, signal transduction, cell cycle, cell proliferation and apoptosis [37]. HBx may exert these effects directly or indirectly and result in HCC. It also induces oxidative stress in the mitochondria, thereby increasing the reactive oxygen species (ROS) levels and inducing the activation of STAT3 and NF-κB [37]. Furthermore, HBx promotes the phosphorylation of STAT3 by activating the IL-6 signaling [38]. HBx induces IL-6 to activate STAT3, and the activated STAT3 cooperatively interacts with hepatocyte nuclear factor 3 (HNF-3) and promotes HBV replication [14]. HBV reactivation can occur after radiotherapy for hepatobiliary malignancies [95], it was observed that IL-6 and radiotherapy synergistically promote the phosphorylation of STAT3, and the phosphorylated STAT3 subsequently forms HNF-3-p-STAT3 heteromeric complexes to its cognate sequence within HBV enhancer 1 [41]. miR-21 is overexpressed in the majority of cancer types and is involved in approximately all tumorigenic processes [96]. miR-21 is upregulated in HCC [97]. HBx activates IL-6 to promote the phosphorylation of STAT3 and upregulate miR-21 expression that leads to the transformation of nontumor hepatocytes [38]. Furthermore, the blockage of STAT3 signaling by shRNAs can promote the cell apoptosis of HBV-positive HCCs and induce cell cycle arrest; HCC cell growth is then inhibited *in vitro* [98]. Using Tet-On inducible cell models, Ching *et al*. [42] discovered that STAT3/NANOG signaling plays a crucial role in enhancing the stemness properties, including self-renewal, tumorigenicity and drug resistance, mediated by C-terminally truncated HBx (HBx-ΔC1). HBx-ΔC1 contributes to the intensification of the aggressive behavior of HCC [99]. NANOG maintains the pluripotency of embryonic stem cells [100] and is identified as an oncogene [101].

Glomerulonephritis, also called HBV-associated glomerulonephritis (HBVGN), is recognized as the most common extrahepatic damage caused by HBV infection. It was found that HBx activates the JAK2/STAT3 signaling pathway and increases the p-STAT3 expression in renal tubular epithelial cells [43]. Subsequently, the activated JAK2/STAT3 signaling increases the ratio between Bax and Bcl-2, which are involved in cell apoptosis [102]. These findings suggest that the JAK2/STAT3 signaling pathway is involved in the process of renal tubular epithelial cell apoptosis in HBVGN caused by HBx [43].

HBsAg, secreted by HBV infected hepatocytes, is the most abundant HBV protein in the peripheral blood of patients with chronic HBV infection (CHB) [103]. A weak HBV-specific T-cell response is usually associated with CHB [104]. It was observed that STAT3 phosphorylation was mediated in an autocrine manner by HBsAg-induced IL-6 in monocytes [39]. HBsAg activates the ERK/IL-6/STAT3 pathway to polarize monocytes toward myeloid-derived suppressor cells (mMDSCs) and impair T cell activation in patients with CHB; mMDSCs can suppress host immune response [105].

In addition, miR-340 expression is downregulated in several types of cancer [106], and this occurrence represses the proliferation, migration and invasion of HCC cells by targeting JAK1 [107]. However, HBV can downregulate the expression of miR-340 to induce STAT3 overexpression [40]. Conclusively, HBV infection activates STAT3 signaling, which benefits HBV replication and promotes the process of HBV-induced HCC.

#### Role of STAT3 in HCV infection

HCV is a leading cause of chronic liver disease. Persistent HCV infection often results in an increased risk of end-stage cirrhosis and HCC [108]. HCV nonstructural proteins, which are localized in the endoplasmic reticulum (ER) membrane, induce ER stress, which leads to the generation of ROS [44]. HCV NS5A protein induces oxidative stress and activates STAT3 and NF-κB [44]. It was also found that HCV RNA activates STAT3 through oxidative stress and induces the activation of tyrosine (JAK and Src) and MAP kinases (JNK and p38 MAPK). Activated STAT3 holds the potential to promote HCV replication [45]. Machida *et al*. [47] further observed that HCV activates STAT3 and DNA damage via ROS.

The HCV core protein also activates STAT3 through the PI3K/Akt and IL-6 pathways [46]. STAT3 activation in the APCs (including monocytes, macrophages and dendritic cells [DCs]) by extracellular HCV core is dependent on the PI3K/Akt pathway and requires newly synthesized IL-6. The activated STAT3 in the APCs may alter APC differentiation, thereby impairing antiviral T-cell response during HCV infection [46]. MDSCs can increase indoleamine 2,3-dioxygenase (IDO) activity to suppress T-cell function [109], and the HCV core protein can induce monocytes to differentiate into MDSCs [110]. Zhai *et al*. [50] found that HCV employed the HCV core to induce MDSC-like monocytes via the TLR/PI3K/AKT/STAT3 signaling pathway. These MDSC-like monocytes inhibits CD4^+ ^T-cell activation and expand CD4^+^CD25^+^Foxp3^+^Tregs by IDO1-induced Trp degradation and accumulation.

HCV infection activates STAT3, which sends a positive feedback to regulate microtubule dynamics. Such regulation in a replicon-based model occurs via a direct sequestration of the microtubule depolymerizing protein STEMN1 to enhance intracellular trafficking of the virus and increase HCV replication [11]. In Huh7 cells, STAT3 promotes HCV replication by upregulating long noncoding RNAs lnc-75K and lnc-IGF2-AS through the augmentation of phosphatidylinositol 4-phosphate (PI4P) expression [48]. Furthermore, HCV RNA could function as miR-122-sponge to upregulate STAT3 expression and inhibit the expression of IFN-α and IFN-β [49].

STAT3 activation by the HCV core in NIH-3T3 cells results in the rapid proliferation and upregulation of Bcl-XL and cylin-D1 [51], while that by HCV NS5A through JAK1-modulated downstream molecules Bcl-XL and p21 expression contributes to HCV-mediated pathogenesis [52]. The HCV core proteins activate NANOG via STAT3 to upregulate cylin-D1 expression and enhance cell proliferation [53]. HCV NS4B induces STAT3 activation through the ER overload response. The activated STAT3 then stimulates the expression of cancer-related target genes, including VEGF, c-myc, MMP-9 and Mcl-1, and promotes human hepatocyte viability [54].

In conclusion, STAT3 can be activated by HCV core, NS4B, NS5B and HCV RNA. Activated STAT3 then contributes to HCV replication by inhibiting host antiviral immune responses and regulating the antiapoptosis and cancer-related genes that may promote the HCV-induced HCC.

### STAT3 in herpesvirus infection

Herpesviruses include α-herpesvirus (HSV and VZV), β-herpesvirus (HCMV, HHV-6 and HHV-7) and γ-herpesvirus (EBV and Kaposi's sarcoma herpesvirus [KSHV]). Meanwhile, some herpesvirus infections are associated with STAT3.

#### Role of STAT3 in HSV infection

HSV infection induces local skin or mucosa membrane lesions, these usually resolve within a few days to several weeks. After being triggered by stress or some stimulators, the virus retreats from neurons where it establishes latency, and is reactivated into the lytic infection. [111]. The latent infection occurs in the sensory or autonomic ganglia and reactivates under physical, hormonal or emotional stress. In the microenvironment of the ganglion, this virus is rapidly activated in the presence of antibody against growth factor (NGF) but not in the concomitant presence of NGF and EGF [112]. Recent studies demonstrated that STAT3 is inhibited in the ganglion culture simultaneously containing NGF and EGF, and the virus can be reactivated. This result indicated that STAT3 plays an essential role in the maintenance of HSV latency, whereas histone acyltransferase (p300/CBP) exerts the opposite effect. Given the lack of direct evidence of blockage of latent virus activation by activated STAT3, Du and colleagues speculate that STAT3 inhibits HSV replication through its own phosphorylation, phosphorylated STAT3 then translocates into the nucleus, interacts with p300/CBP, and then activates certain genes, such as the neuroprotective-related genes *Bcl-2, Bcl-XL, IAP2, Pim-1*, neurodegenerative gene *GAP-43*, and neurodevelopmental gene *GFPAF*, to maintain the latent state of HSV [80]. Another study reported that the HSV-1 infection of neural progenitor cells results in the loss of neuronal precursors, although this loss can be prevented by the addition of microglia or conditioned media from neural progenitor cells/microglia co-cultures through the IL-6- induced STAT3 cascade [81].

To identify how STAT3 regulates the innate immune responses during the early phase of HSV-1 lytic infection, Hisa *et al*. [82] utilized the myeloid-specific STAT3-knockout mice. They found that STAT3-knockout bone marrow-derived macrophages (BMMs) decreased the levels of IFN-α and interferon-stimulated genes (ISGs) *in vivo* upon HSV-1 infection. The STAT3-knockout mice were more susceptible to HSV-1 than the wild-type mice and failed to expand the CD8^+^ conventional DC population upon HSV-1 infection accompanied by the impaired NK and CD8^+ ^T-cell activation. This report demonstrated that myeloid-specific STAT3 deletion causes defects in multiple aspects of the immune system in response to HSV-1. These findings suggested that STAT3 has a protective role at the early stage of systemic HSV-1 infection [82].

#### Role of STAT3 in VZV infection

VZV causes varicella during primary infection and zoster upon reactivation. VZV triggers STAT3 phosphorylation in infected cells *in vitro* and in human skin xenografts in SCID mice *in vivo*. STAT3 activation induces the antiapoptotic protein survivin and promotes VZV infection via increasing the survivin expression and inhibition of apoptosis [13]. Another work demonstrated that VZV infection can upregulate miR-21 to activate the STAT3 pathway and promote VZV replication in MeWo and human embryonic lung fibroblast cell lines [55].

#### Role of STAT3 in HCMV infection

HCMV causes an asymptomatic latent infection in healthy individuals. However, HCMV infection sometime leads to severe diseases involved in immune deficiency, especially in patients with AIDS and in immunocompromised solid organ and bone marrow allograft recipients. HCMV primarily utilizes unphosphorylated STAT3 to promote HCMV DNA replication [56]. HCMV infection disrupts the IL-6-induced phosphorylation of STAT3 and inhibition of a subset of IL-6/STAT3-regulated gene expression, including SOCS3. A 72 kDa immediate early1 (IE1) protein of HCMV associated with STAT3 is necessary to localize STAT3 to the nucleus during infection; this protein diminishes HCMV genome replication [56]. The HCMV infection of HepG2 and primary human hepatocyte cells results in the activation of the IL-6/JAK/STAT3 pathway, which enhances the HepG2 tumor sphere formation and the transformation of primary human hepatocyte cells. HCMV infection appears to be involved in the genesis of HCC [57].

#### Role of STAT3 in EBV infection

EBV mainly invades B lymphocytes and exhibits an affinity to pharyngeal and glandular cells, which are closely correlated to Burkitt's lymphoma, Hodgkin's lymphoma, NK/T-cell lymphoma, post-transplant lymphoproliferative diseases and nasopharyngeal cancer (NPC). EBV LMP1 stimulated STAT3 signaling in a human NPC cell line. LMP1 can phosphorylate STAT3 at Tyr705 by activating JAK3 and phosphorylate STAT3 at Ser727 by activating ERK. STAT3-p705 is correlated with NPC stages III and IV; this observation suggests that STAT3 plays an important role in NPC invasion and metastasis [58]. EBV LMP1 C-terminal-activating region 1 (CTAR1) specifically induces EGFR expression, which occurs at high levels in NPC. This induction requires NF-κB mediated by the TRAF signaling pathway [59]. In the study of Kung *et al*. [59], LMP1-CTAR1 expression increased the phosphorylation of STAT3 at serine and tyrosine. The serine-phosphorylated STAT3 binds to sites within the Bcl-3 promoter and intron 2, thus increasing the nuclear Bcl-3 levels. Binding with p50 homodimer, the increased Bcl-3 forms a unique complex of NF-κB that positively regulates EGFR expression. The researchers further studied the effects of LMP1 on EGFR and STAT3 activation and found that LMP1–CTAR1 activates STAT3 and EGFR in a serum-independent manner with the constitutive serine phosphorylation of STAT3. PKCδ is the kinase responsible for activating STAT3. Inhibition of PKCδ also inhibited the constitutive phosphorylation of EGFR and ERK [60]. Apart from LMP1, Epstein–Barr nuclear antigen 2 (EBNA2) also augmented the transcriptional activity of STAT3 by enhancing the STAT3–DNA binding ability. EBNA2 synergistically acted on STAT3 activation with LMP1 [61]. In addition, EBV LMP2A activates the PI3K/BTK pathway to phosphorylate STAT3 in B-cell tumors and increases IL-10 expression [62].

Constitutive STAT3 activation is reportedly correlated with advanced clinical staging in NPC [58]. Lui *et al*. [63] demonstrated that EBV-induced STAT3 activation directly contributes to the intrinsic invasiveness of NPC cells. STAT3–Tyr705 is mainly detected in NPC tumors. They utilized the NPC cell line model HONE-1 and found that EBV induced a high level of STAT3–Tyr705. SiRNA targeting STAT3 inhibited the invasion and cell growth. Conversely, the activation of STAT3 enhanced the invasiveness and proliferation, as well as increased the expression of markers of mesenchymal status, proliferation and invasion. Wang *et al*. [64] found that LMP1 activates STAT3 through the JAK3/STAT3 and MEK1/ERK1/2 pathways, and the activation of STAT3 induces VEGF, and finally enhances NPC cell migration. LMP1-activated EGFR and STAT3 directly targets the cyclinD1 promoter and upregulates the cyclin D1 promoter activity and mRNA level in CNE 1 cells [65]. Meanwhile, LMP1 downregulates miR-204, which functions as a tumor suppressor by activating STAT3 to promote EBV-positive C666-1 cell invasion and metastasis [66].

EBV persists as a lifelong infection by maintaining the latent phase of its life cycle within B lymphocytes. Upon infecting primary B cells, EBV must initially drive cell proliferation to establish latency. The exposure of healthy subject-derived B cells to EBV early resulted in the activation of STAT3, and the activation is necessary for the subsequent survival and proliferation of EBV-infected cells past the S phase of the cell cycle. However, B cells derived from patients with autosomal dominant hyper-IgE syndrome (AD-HIES) are impaired in their susceptibility to EBV-driven growth transformation. Patients with AD-HIES harbor a dominant negative mutation in their STAT3 gene [113].

The lytic activation of EBV is essential to the life cycle of the virus and to most EBV-related diseases. Antiviral agents that eliminate EBV-positive tumor cells induce the viral lytic cycle. In cell culture, HDAC inhibitors can induce EBV to enter the lytic cycle. However, only a fraction of cells enter the lytic cycle; the remainder of the population is refractory in this process. Daigle and his colleagues separated the lytic cells from refractory Burkitt lymphoma-derived HH514-16 cells after treatment with an HDAC inhibitor. They found that STAT3, Fos and IL-8 were upregulated in the refractory cell population, whereas IL-6 was upregulated in the lytic population. Examination of single cells revealed that high levels of STAT3 are strongly associated with the refractory state [67]. The group further investigated whether STAT3 controls the susceptibility to EBV lytic activation and found that the levels of activated STAT3 regulated the susceptibility to EBV lytic activation. Further experiments confirmed that the increased expression of three KRAB-ZFPs and a histone methyltransferase were likely transcriptionally upregulated by STAT3 in refractory cells. The link between STAT3 and lytic susceptibility shows that STAT3 may favor the persistence of tumor cells by limiting lytic activation in EBV-induced tumors [68]. Koganti *et al*. [69] further observed that STAT3 functions via cellular poly (C)-binding protein 2 (PCBP2) to regulate the lytic susceptibility.

#### Role of STAT3 in KSHV infection

KSHV is the infectious cause of Kaposi's sarcoma, primary effusion lymphoma and multicentric Castleman's disease. STAT3 is activated in KSHV-infected endothelial cells by activating gp130 and JAK2. The activation persists for as long as the latent infection is maintained. The persistent activation of STAT3 may play a critical role in the viral pathogenesis of Kaposi's sarcoma, as well as primary effusion lymphoma [70]. Muromoto *et al*. [114] discovered that KSHV-encoded latency-associated nuclear antigen induces the activation of STAT3.

### STAT3 in respiratory virus infection

Respiratory viruses mainly cause respiratory tract lesions and other organ lesions. Research has shown that IL-6 is an important factor regulating host susceptibility to respiratory tract infection. IL-6 plays an important role in host defense and the prevention of lung injury against pathogens. Given that IL-6 activates the JAK/STAT3 signaling cascade, STAT3 contributes to the pathogenic process of respiratory viruses.

#### Role of STAT3 in Influenza A virus infection

Influenza A virus (IAV) is a highly contagious single-stranded RNA virus that infects both the upper and lower respiratory tracts of humans and is considered a major worldwide public health problem. Although seasonal infections with the most common influenza virus strains (e.g., H3N2) can be resolved typically, infection continues to cause a high rate of mortality [115]. IAV H5N1 and H1N1 nonstructural protein 1 (NS1) can disrupt IFN signaling by inhibiting IFNAR expression and upregulating the levels of the JAK/STAT inhibitors SOCS1 and SOCS3. This process leads to a notable reduction of IFN-inducible STAT3 phosphorylation and DNA binding [83]. Mahony and colleagues found that STAT3 is required to inhibit IAV replication. STAT3 is specifically required for inducing a subset of IFN-α-driven ISGs, including PKR, OAS2, MxB and ISG15 [84]. Another research reported that highly pathogenic avian influenza, H5N1 delays apoptotic responses by activating STAT3. H5N1 and H1N1 inhibit the activation of STAT3, but H5N1 infection leads to the further activation of STAT3, which probably contributes to the pathogenesis of severe influenza disease [85]. However, the detailed mechanism about how STAT3 regulates the induction of apoptosis in influenza virus requires further investigation.

#### Role of STAT3 in mumps virus & measles virus infections

Mumps virus infection causes parotitis, meningitis and orchitis. Mumps virus encodes a V protein that can assemble into an ubiquitin ligase complex that causes the polyubiquitylation and proteasome-mediated degradation of cellular STAT1 and STAT3 proteins, to modulate host innate and adaptive antiviral responses [86,116]. Furthermore, the mumps virus V protein prevents responses to IL-6 and v-Src, both of which are STAT3 signaling triggers [86].

Measles virus infection is characterized by the virus-induced immune suppression that increases the susceptibility to opportunistic infections. Similarly, the measles virus V protein also interferes with STAT3 activation and provides several general or tissue-specific replication advantages to the virus [87].

#### Role of STAT3 in other respiratory infection

SARS-CoV infection causes SARS, which may be mediated by the viral replication in target cells and immune responses. SARS-CoV infection can activate p38 MAPK, ERK1/2 and JNK pathways in Vero E6 cells, and the p38 MAPK pathway induces STAT3 dephosphorylation at Tyr705 to promote SARS-CoV replication [88].

Human metapneumovirus (hMPV) infection can result in bronchiolitis or pneumonia and can exacerbate chronic obstructive pulmonary disease. hMPV can attenuate the IL-6-mediated JAK/STAT3 signaling cascade in lung epithelial cells. It also interrupts the IL-6-induced activation and nuclear translocation of STAT3 by inhibiting the phosphorylation of JAK2, and further regulates the STAT3 downstream genes for apoptosis, cell proliferation and differentiation [89]. However, the identity of the cellular target for the hMPV inhibition of the IL-6 signaling pathway remains unknown; the target may be the IL-6 receptor complex.

Reproductive and respiratory syncytial virus (RSV) infection causes airway inflammation and exacerbates asthma. Respiratory syncytial virus can activate STAT3 via IL-6, and the activation is necessary for the viral early gene activation and successful infection of epithelial cells [71]. Porcine reproductive and respiratory syndrome virus is an economically important viral disease in the swine industry. The viral nonstructural protein 5 C-terminal domain induces STAT3 degradation by increasing its polyubiquitination level and shortening the half-life [90], which could lead to the suppression of a broad spectrum of cytokines and growth factors to thwart host antiviral responses and allow virus replication.

### STAT3 in enterovirus infection

Coxsackievirus B3 infection induces a severe inflammation during the acute phase of the resultant viral myocarditis. STAT3 signaling plays a protective function in Coxsackievirus B3-induced myocarditis [91]. We also observed that enterovirus 71 infection decreases STAT3 phosphorylation but increases miR-124 expression, which inhibits STAT3 signaling by directly targeting STAT3 mRNA or indirectly via targeting the upstream IL-6R [92]. These findings suggested that STAT3 has antiviral activity against EV 71 infection. However, EV 71 could dysregulate miR-124 to evade the host immune response.

### STAT3 in retrovirus infection

HIV infection induces AIDS. HIV-1 Nef mediates STAT3 activation in immature DCs [117]. Percario *et al*. [72] found that Nef C-terminal flexible loop activates STAT3 through the release of soluble factor(s), including NF-κB, MIP-1α and IL-6. In gastric epithelial cells, HIV also activates STAT3, RelA and IL-6 [73]. HIV-1 gp120 resulted in the production of IL-6 via MAPK/NF-κB pathway; IL-6 in turn activated STAT3. Concomitantly, gp120 promoted an early activation of STAT3 that further contributed to IL-6 induction in immature monocyte-derived DCs. HIV-1 gp120-induced dysregulation of IL-6/STAT3 may account for the impairment of DC function observed upon HIV exposure [74].

Human T-cell leukemia virus type-1 (HTLV-1) predominantly infects T lymphocytes, causing two distinct diseases, adult T-cell leukemia/lymphoma and HTLV-1 associated myelopathy/tropical spastic paraparesis. HTLV Tax protein upregulates IL-6R and sIL-6R, which may contribute to the proliferation of HTLV-1-infected T cells through the activation of STAT3 and affect the malignant growth and transformation of T cells induced by HTLV-1 [75]. IL-10-mediated signals promote the proliferation of HTLV-1-infected cell through STAT3 and IRF4 pathway [76].

### STAT3 in other virus infection

In DCs, Dengue virus type 2 nonstructural 1 (NS1) protein interacts with STAT3β protein, which may influence the pathological changes observed in dengue fever, dengue hemorrhagic fever and dengue shock syndrome [77]. Dengue virus infection induces chemokine production by activating the JAK/STAT3 signaling pathway in HepG2 cells [78]. Theiler's murine encephalomyelitis virus results in the vigorous production of IL-6, which promotes the generation of Th17 cells. IL-6 and IL-17 synergistically promote viral persistence by protecting virus-infected cells from apoptosis and CD8^+^ T cell-mediated target destruction by activating STAT3 and NF-κB [79]. Rabies virus P protein interacts with STAT3 and inhibits the STAT3 nuclear accumulation and gp130-dependent signaling [93]. Simian virus 40 large tumor antigen (TAg) increases STAT3 tyrosine phosphorylation, DNA binding and the transcriptional activity. STAT3 is required for TAg-mediated neoplastic transformation [118]. STAT3 is also required for the Epo-independent growth of Friend erythroleukemia virus-infected cells. The activation of STAT3 by Stk receptor tyrosine kinase is mediated by a novel STAT3 binding site in Gab2 [119].

## Conclusion

STAT3 plays an important role in viral infection. It can be activated by virus-encoded proteins, such as HBx protein, HCV core protein, NS5A, EBV LMP1 and HIV Nef. However, STAT3 activation can also be inhibited by some viruses or viral proteins including IAV NS-1, hMPV and SARS-CoV (Table 1 & Figure 3). The dysregulation of STAT3 by these viruses can cause activation or inhibition of downstream signaling, and results in pathogenesis. STAT3 signaling also interacts with other host factors in a transcriptional activity-independent way to regulate antiviral and inflammatory responses. The role of STAT3 is well documented in several oncoviruses, but its function in other viruses remains unclear. However, on the basis of the present data, we conclude that the host STAT3 signaling responses to virus infections are complicated and it would be regulated in a virus-specific manner and even with a stage-specific characteristic in the virus life cycle. Given that STAT3 activation could be triggered by different virus proteins or virus-induced host factors, and specific kinase members would be recruited to activate STAT3 after different virus infection, the downstream target genes regulated by STAT3 must be distinctive. Therefore, STAT3 has the virus-specific effects on the virus life cycle and contributes to the viral pathogenesis.

## Future perspective

From the literature collected in this review, it clearly shows that STAT3 plays an important role in the virus life cycle and in viral pathogenesis. However, the key viral and host factors that promote the activation or inhibition of STAT3 signaling after viral infection are largely unknown except several viruses listed in Table 1. If more viruses were identified to trigger or inhibit STAT3 activation by their viral proteins or their genomes, it would be helpful to understand the molecular mechanism of interaction between virus and host STAT3 signaling. Specifically, viruses with a close relation in taxonomy might provide the general conception.

Further in depth studies are necessary to determine the mechanisms by which viruses exploit STAT3 for their life cycle. In the early and late stage of virus replication, when and how viruses activate STAT3 signaling by different viral proteins or genome? For examples, HBV activated STAT3 could be induced by HBx and HBsAg, and HCV activated STAT3 could be triggered by HCV core, NS4B, NS5B and HCV RNA (Table 1). When and where would the activation of STAT3 be induced by the viral proteins or their genomes? Is it a viral replication stage-specific or tissue specific event?

Moreover, STAT3 exerts the contradictory effects on the viral infection, antiviral to some virus and proviral to others. What is key factor to determine the switch between the proviral and antiviral function of STAT3? Further study is needed for the identification of the factors that drive STAT3 signaling to play antiviral or proviral role.

Furthermore, in the viral pathogenesis, further studies are also needed for the identification of the mechanisms underlying host STAT3 function in an immune-protective or proinflammatory response. On this issue, many questions need to be resolved. For example, how viruses regulate STAT3 to respond to IFN? STAT3 negatively or positively regulates type 1 IFN response depending on the virus type involved? How viruses regulate STAT3 response to different cytokines, such as IL-6, IL-10 and IFNs, especially, when these cytokines induced by the virus in different tissues?

Conclusively, a comprehensive analysis to dissect the role of STAT3 in viral infections in the future can provide valuable insights into the viral pathogenesis and development of novel antiviral therapies.

**Executive summary**

- As a STATs family member, STAT3 plays a central regulatory role in cell proliferation, differentiation, apoptosis and oncogenesis, and is also involved in the process of cellular immune responses and inflammation.
- STAT3 signaling is mainly activated by the IL-6 and IL-10 family cytokines and growth factor receptors. STAT3 phosphorylated at Tyr705 is required for dimerization to activate STAT3 signaling, which would be inhibited by suppressor of cytokine signaling, protein inhibitor of activated STAT and protein tyrosine phosphatase.
- STAT3 has a proviral function in several viral infections. However, in some circumstances, STAT3 has an antiviral activity in other viral infections. The activation or inhibition of STAT3 signaling is a virus-specific event.
- HBV infection activates STAT3 through HBx and hepatitis B surface antigen. HBx increases  reactive oxygen species, induces IL-6/STAT3/NANOG signaling, and regulates some miRNAs to promote the HBV-induced hepatocellular carcinoma. hepatitis B surface antigen activates the ERK/IL-6/STAT3 pathway to regulate monocyte toward myeloid-derived suppressor cells and T-cell activation.
- HCV NS4B, NS5B and HCV RNA activate STAT3 signaling through reactive oxygen species; HCV core activates STAT3 through the PI3K/Akt and IL-6 pathways. STAT3 activation contributes to HCV replication by inhibiting host antiviral immune responses and regulating the antiapoptosis and cancer-related genes that may promote the HCV-induced hepatocellular carcinoma.
- STAT3 plays a protective role in the early stage of HSV-1 infection. However, STAT3 activation contributes to maintain HSV-1 latency by inhibiting p300/CBP activity.
- EBV activates STAT3 signaling to inhibit lytic activation through PCBP2, and upregulates EGF receptor, JAK3 and STAT3 pathways for cancer cell proliferation, invasion and metastasis by LMP1, LMP2A and EBNA2.
- STAT3 increases HCMV replication and promotes HCMV-infected cell transformation. Varicella zoster virus activates STAT3 for its replication dependent on the increase of survivin, this occurrence also required for Kaposi's sarcoma herpesvirus-induced malignant transformation.
- Activation of STAT3 signaling shows antiviral activity against most of respiratory viruses, including influenza virus, measles virus, mumps virus, severe acute respiratory syndrome coronavirus, human metapneumovirus and porcine reproductive and respiratory syndrome virus, but reproductive and respiratory syndrome virus.
- STAT3 signaling responses to virus infection are complicated in a virus-specific manner and even in a stage-specific manner in the virus life cycle. It needs further investigation why STAT3 has been distinctively regulated by the different viruses. Specifically, to some viruses within a close branch in taxonomy.
